# Antinociceptive effect of geranylgeraniol and 6α,7β-dihydroxyvouacapan-17β-oate methyl ester isolated from *Pterodon pubescens *Benth

**DOI:** 10.1186/1471-2210-10-1

**Published:** 2010-01-07

**Authors:** Humberto M Spindola, Leila Servat, Carina Denny, Rodney AF Rodrigues, Marcos N Eberlin, Elaine Cabral, Ilza MO Sousa, Jorge Y Tamashiro, João E Carvalho, Mary A Foglio

**Affiliations:** 1 CPQBA- State University of Campinas, P.O. Box 6171, 13083-970 Campinas-SP, Brazil; 2 Department of Pharmacology, Anesthesiology and Therapeutics, Faculty of Dentistry, State University of Campinas, P.O. Box 52, 13414-903 Piracicaba-SP, Brazil; 3 Biology Institute, State University of Campinas, P.O. Box 6109, 13083-970 Campinas-SP, Brazil; 4 Thomson Mass Spectrometry Laboratory, Chemical Institute, State University of Campinas, P.O.BOX 6154, 13084-862 Campinas-SP, Brazil; 5 Chemistry Institute - São Paulo University, P. O. Box 26.077, 05513-970 São Paulo - SP, Brazil

## Abstract

**Background:**

*Pterodon pubescens *Benth seeds are commercially available in the Brazilian medicinal plant street market. The crude alcoholic extracts of this plant are used in folk medicine as anti-inflammatory, analgesic, and anti-rheumatic preparations. The aim of this study was to evaluate the contribution of geranylgeraniol (C1) and 6α, 7β-dihydroxyvouacapan-17β-oate methyl ester (C2) isolated from *Pterodon pubescens *Benth. to the antinociceptive activity of the crude extract.

**Results:**

Compounds C1 and C2 demonstrated activity against writhing with intraperitoneal (i.p.) and oral (p.o.) routes, capsaicin (i.p. and p.o.), glutamate (i.p.), and in the hot-plate (p.o.) tests, demonstrating their contribution to the antinociceptive activity of crude *Pterodon pubescens *Benth extracts. The observed activity of compounds C1 and C2 may be related to vanilloid receptors VR1, and/or glutamate peripheral receptors. In hot-plate model, the antinociceptive activity was maintained when naloxone chloride (opioid antagonist) was administered prior to treatment with compounds suggesting that C1 and C2 (p.o.) do not exert their antinociceptive effects in the hot-plate test via opioid receptors. The findings presented herein also suggest that compounds within the crude *Pterodon pubescens *Benth. extract may exert a synergistic interactive effect, since the crude extract (300 mg.kg^-1^) containing lower concentrations of compounds C1 (11.5%- 34.6 mg. kg^-1^) and C2 (1.5% - 4.7 mg.kg^-1^) gave statistically the same effect to the pure compounds when tested separately (C1 = C2 = 300 mg.kg^-1^) in writhing experimental model with p.o. administration. Further studies will be undertaken to establish more specifically the mechanisms of action for compounds C1 and C2. Possible synergistic interactions will be evaluated employing the Isobole method.

**Conclusion:**

These results allowed us to establish a relationship between the popular use of *Pterodon pubescens *seeds for pain relief and the activity of two major compounds isolated from this species which demonstrated antinociceptive activity. Various "*in vivo" *experimental models corroborate the folk use of this species for different pain and inflammation disorders.

## Background

### Pterodon pubescens

Benth. (Leguminosae), known as sucupira, is widespread throughout the Brazilian states of Goiás, Minas Gerais and São Paulo. Sucupira seeds are commercially available in the Brazilian medicinal plant market. The crude alcoholic extracts of this plant are used in folk medicine as anti-inflammatory, analgesic and anti-rheumatic preparations [1,2]. Phytochemical studies of the *Pterodon *genus have shown the presence of alkaloids, isoflavones and diterpenes. Furanditerpenes were identified and isolated from *Pterodon *fruits [3-7]. Studies have suggested that furanditerpenes possessing the vouacapan skeleton contribute to the anti-inflammatory and antinociceptive properties of *Pterodon pubescens *seed oil [8-12]. Diterpenes 6α-hydroxyvouacapan-7β-17β-lactone and 6α, 7β-dihydroxyvouacapan-17β-oate methyl ester, found in *P. emarginatus *and *P. polygalaeflorus *seeds were previously reported to be associated with the anti-inflammatory activity of these species [8]. Herein we report the antinociceptive activity of 6α, 7β-dihydroxyvouacapan-17β-oate methyl ester and geranylgeraniol isolated from *Pterodon pubescens *Benth. when evaluated in writhing, capsaicin, glutamate and hot-plate animal experimental models.

## Results and Discussion

**Figure 1:**
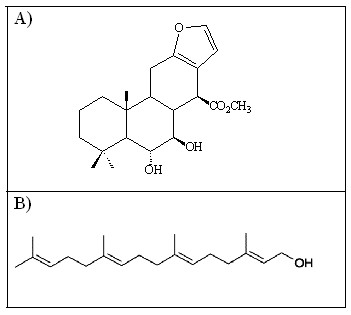
**Chemical structures of compounds A) 6α, 7β-dihydroxyvouacapan-17β-oate methyl ester (C2), and B) Geranylgeraniol (C1)**.

Some authors have reported the antinociceptive activity of the crude extract and fractions obtained from *P. pubescens *and established a relationship with anti-inflammatory activity [10,12]. This report evaluated for the first time the contribution of geranylgeraniol (C1) and 6α, 7β-dihydroxyvouacapan-17β-oate methyl ester (C2), isolated from *P. pubescens *(Fig. 1), to the antinociceptive activity using various experimental models to evaluate a distinct pain modulation.

Calixto et al [13] demonstrated the antiplatelet activity of geranylgeraniol attributed to cyclooxygenase enzyme inhibition, but did not mention data on antinociceptive activity. Some vouacapan compounds have been suggested to have a relationship with *P. pubescens' *antinociceptive activity. Nevertheless pain modulation by this species has never been reported, being described herein for the first time. The following screening results demonstrated the activity and general mechanisms involved in antinociception caused by geranylgeraniol (C1) and 6α, 7β-dihydroxyvouacapan-17β-oate methyl ester (C2).

The most relevant additional findings of the present work are that, (i) compounds C1 and C2 may present synergistic activity; (ii) both intraperitoneal (i.p.) and oral (p.o.) treatment of compounds C1 and C2 reduced reactivity to the writhing test demonstrating differences in potency related to the route of administration; (iii) both compounds C1 and C2 demonstrated possible activity related to vanilloid receptors and/or glutamate peripheral receptors, with C2 being more potent by the i.p. route; (iv) the antinociceptive activity of compounds C1 and C2 (p.o.) do not appear to exert their antinociceptive effects in the hot-plate test via opioid receptors.

Initially, the open field test was performed in order to exclude the possibility that the antinociceptive action of geranylgeraniol (C1) and 6α, 7β-dihydroxyvouacapan-17β-oate methyl ester (C2) could be related to non-specific disturbances in the locomotor activity of the animals. Treatment with compounds C1 and C2 (30 mg.kg^-1^, i.p.) did not cause any significant change in the ambulation of mice when tested in the open field. However, pentobarbital (35 mg.kg^-1^, i.p.) significantly (p < 0.001) reduced the locomotor activity of animals in this test. The mean number of crossings was 48.2 ± 2.2, 13.7 ± 6.1, 50 ± 4.4 and 42 ± 3.6 for vehicle, pentobarbital, C1 and C2, respectively.

After these results, the acetic acid writhing test model was employed. This is a convenient stimulus assay for screening, because the intensity of response depends on the interaction of several factors, neurotransmitters and neuromodulators that determine nociception, such as kinines, acetylcholine, substance P and prostaglandins. Therefore, this model is responsive to analgesic substances possessing with the most varied action mechanisms [14,15], being sensitive to drugs with analgesic activity such as aspirin, kinin receptor antagonists (bradykinin, kallidin or T-kinin) and opioid analgesics with central or peripheral action [16,17]. This model permitted evaluation of antinociceptive activity caused by both neurogenic and/or inflammatory pain. This assay was used during the initial studies with compounds C1 and C2 in order to establish differences in potency using different routes of administration.

The antinociceptive activity of compounds C1 and C2 were compared with the dichloromethane extract (EB) (p.o.) in the writhing test using 300 mg.kg^-1 ^doses. The reductions in the number of abdominal constrictions were 62%, 64% and 66% for EB, C1 and C2 respectively (p ≤ 0.001) (not shown). These results suggested a possible synergistic activity among compounds C1 and C2 [these compounds are only at concentration: C1 (11.56%- 34.6 mg. kg^-1^) and C2 (1.58% - 4.7 mg.kg^-1^) in the EB fraction]. Accordingly, dose-response curves for i.p. and p.o. administered compounds were determined in the writhing test to calculate ED_50 _values and possible differences caused by administration routes.

**Figure 2:**
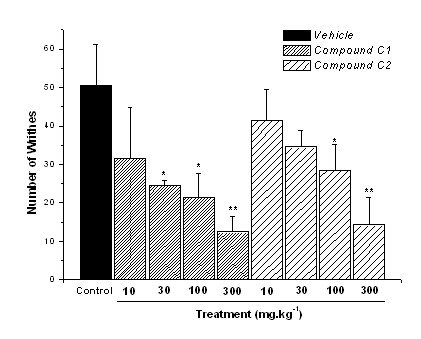
**Abdominal constrictions induced by acetic acid (0.8% in saline) in mice previously administered (60 min) p.o. with control vehicle (10 mL.kg^-1^), or compounds C1 and C2 (dose-response)**. Results expressed as mean ± SEM of up to 8 animals for experimental groups (*p ≤ 0.05; **p ≤ 0.01).

**Figure 3:**
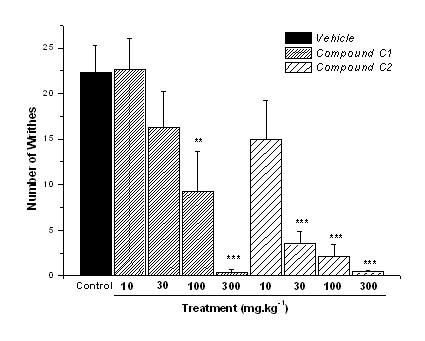
**Abdominal constrictions induced by acetic acid (0.8% in saline) in mice previously administered (30 min) i.p. with control vehicle (10 mL.kg^-1^), or compounds C1 and C2 (dose-response)**. Results expressed as mean ± SEM of up to 8 animals for experimental groups (**p ≤ 0.01; ***p ≤ 0.001).

The p.o. administration of compound C1 showed dose-related activity reducing by 51%, 57%, and 75% (p ≤ 0.01) the abdominal constrictions with 30 mg.kg^-1^, 100 mg.kg^-1^, and 300 mg.kg^-1 ^doses respectively, presenting ED_50 _= 26.7 mg.kg^-1 ^(Fig. 2). The same treatment with compound C2 reduced constrictions by 43% (100 mg.kg^-1^) and 71% (300 mg.kg^-1^) with ED_50 _= 35.6 mg.kg^-1^. These results showed that compound C1 was more potent than C2 when given by p.o. administration. The dose-related activity for compounds administered i.p. (systemic route) in the writhing test, demonstrated that compound C1 reduced constrictions by 58% and 98% (p ≤ 0.001) with 100 mg.kg^-1 ^and 300 mg.kg^-1 ^doses respectively (ED_50 _= 22.4 mg.kg^-1^) (Fig. 3). Compound C2 reduced constrictions by 84%, 90%, and 98% with 30 mg.kg^-1^, 100 mg.kg^-1^, and 300 mg.kg^-1 ^doses respectively, showing ED_50 _= 11.5 mg.kg^-1^, more potent than C1. In the i.p. tests, the numbers of abdominal constrictions of the control group were fewer compared to the control group of the p.o. route, maybe caused by injection-stress of the animals.

In order to evaluate the antinociceptive response of compounds C1 and C2 in neurogenic pain caused by direct chemical stimulation on nociceptors, the capsaicin test was evaluated. The active principle, Capsaicin, from *Capsicum *genus peppers, is the painful substance used to determine if compounds possessing antinociceptive activity act by the vanilloid VR1 receptors. The animal response is caused by release of neuropeptides, such as substance P, neurokinines, somastotastin, peptide related to the calcitonin gene (CGRP) with participation of nociceptive afferent C fibers, and in part, A fibers [18,19]. The induced nociceptive process with capsaicin is related to the activation of the tachykininergic system and seems to be mediated by activation of a specific receptor, whose presence has been confirmed with capsazepine, a competitive vanilloid antagonist. Evidence also suggests that bradykinin (BK) acting through B_2 _receptors seems to be involved in the neurogenic nociception caused by capsaicin in mice [20].

**Figure 4:**
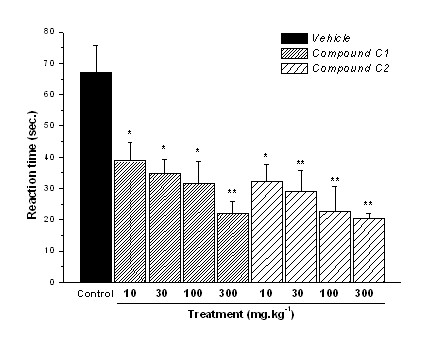
**Reactivity time to the intraplantar application of capsaicin (1.6 μg.paw^-1^) in the hind paw of mice previously treated (30 min) i.p. with control vehicle (10 mL.kg^-1^), or compounds C1 and C2 (dose-response curve)**. Results expressed as mean ± SEM of up to 8 animals for experimental groups (* p ≤ 0.05; ** p ≤ 0.01).

**Figure 5:**
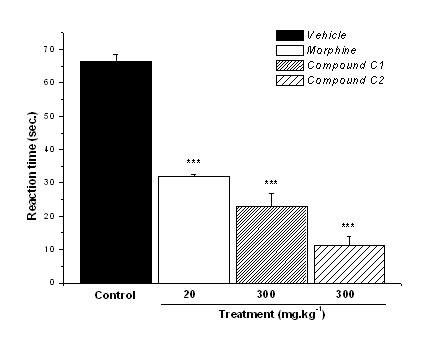
**Reactivity time to the intraplantar application of capsaicin (1.6 μg.paw^-1^) in the hind paw of mice previously treated (30 min) p.o. with control vehicle (10 mL.kg^-1^), morphine (20 mg.kg^-1^) or compounds C1 and C2 (300 mg.kg^-1^)**. Results expressed as mean ± SEM of up to 8 animals for experimental groups (***p ≤ 0.001).

In this test, i.p. administration of compound C1 reduced by 43%, 48%, 58%, and 67% (p ≤ 0.01) with 10 mg.kg^-1^, 30 mg.kg^-1^, 100 mg.kg^-1^, and 300 mg.kg^-1 ^doses respectively (ED_50 _= 34.1 mg.kg^-1^) (Fig. 4). Compound C2 showed dose-related activity too, reducing by 51%, 56%, 66%, and 69% (p ≤ 0.01) the reaction time, with 10 mg.kg^-1^, 30 mg.kg^-1^, 100 mg.kg^-1^, and 300 mg.kg^-1 ^doses respectively (ED_50 _= 27.8 mg.kg^-1^). These results showed that the antinociceptive activity of compounds C1 and C2 may be related to the tachykininergic system or vanilloid VR1 receptors. The same experimental model was tested with p.o. administration of compounds C1 (300 mg.kg^-1^) and C2 (300 mg.kg^-1^) employing morphine (20 mg.kg^-1^) as positive control, in order to evaluate the activity of compounds on this route (Fig. 5). Compound C2 reduced reaction time by 82% whereas C1 reduced by 65%, with the positive control demonstrating reduction by 52% (p ≤ 0.001). This assay demonstrated that compound C2 was more potent in neurogenic pain modulated by vanilloid receptors than compound C1, corroborating the previous result with intraperitoneal administration.

Once we had demonstrated the activity of compounds geranylgeraniol (C1) and 6α,7β-dihydroxyvouacapan-17β-oate methyl ester (C2) with the writhing and capsaicin tests, we considered the glutamate test. This model is based on the activation of peripheral glutamate receptors responsible for several types of pain sensation. The nociceptive response induced by glutamate appears to involve peripheral, spinal and supraspinal sites of action and is mediated by both NMDA and non-NMDA receptors as well as by the release of nitric oxide or by some nitric oxide-related substances. Nitric oxide inhibitors and both NMDA and non-NMDA receptor antagonists, and other drugs have been previously reported to inhibit the acetic-acid and capsaicin- induced nociceptive response [21].

**Figure 6:**
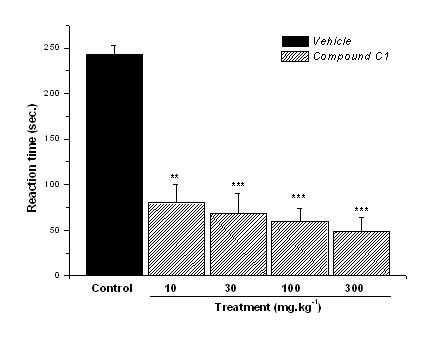
**Reactivity time to the intraplantar application of glutamate (10 μmol.paw^-1^) in the hind paw of mice previously treated (30 min) i.p. with control vehicle (10 mL.kg^-1^) or compound C1 (dose-response curve)**. Results expressed as mean ± SEM of up to 8 animals for experimental groups (**p ≤ 0.01 ***p ≤ 0.001).

**Figure 7:**
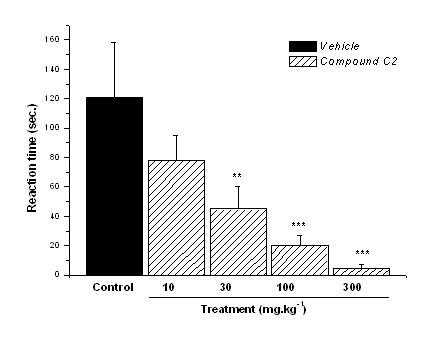
**Reactivity time to the intraplantar application of glutamate (10 μmol.paw^-1^) in the hind paw of mice previously treated (30 min) i.p. with control vehicle (10 mL.kg^-1^) or compound C2 (dose-response curve)**. Results expressed as mean ± SEM of up to 8 animals for experimental groups (**p ≤ 0.01 ***p ≤ 0.001).

Our results showed that i.p. administration of compounds C1 and C2 produced a significant dose-related inhibition of the nociceptive response caused by intraplantar injection of glutamate into the mouse's hind paw. In this test, the p.o. route was not evaluated because of possible changes of pharmacokinetic, metabolic or distribution parameters, which could disrupt the interaction among compounds and receptors (considering the wide receptors distribution). Compound C1 (i.p.), with 10 mg.kg^-1^, 30 mg.kg^-1^, 100 mg.kg^-1^, and 300 mg.kg^-1 ^doses reduced reaction time by 67%, 72%, 75%, and 80% respectively (p ≤ 0.001), corresponding to on ED_50 _= 57.4 mg.kg^-1 ^(Fig. 6). Compound C2 (i.p.) showed ED_50 _= 35.1 mg.kg^-1^, reducing by 62%, 83%, and 96% the reaction time with 30 mg.kg^-1^, 100 mg.kg^-1^, and 300 mg.kg^-1 ^doses (p ≤ 0.001) respectively (Fig. 7), again more potent than C1 in peripheral neurogenic modulation. Thus, these previous findings and the present results suggested that the antinociceptive action of compounds C1 and C2 in the acetic acid, capsaicin, and glutamate tests could be the result of both the inhibition of NOS and the blockade of glutamate receptors. Further studies are being undertaken to confirm receptors involved on this activity.

Compounds C1 and C2 were evaluated in the hot-plate thermal nociception model by p.o. route. This route was chosen based on preliminary set of experiments which showed to induce less stress to animals. The hot-plate test is a neurogenic-modulated model that produces, at constant temperature, two kinds of behavioral response, which are paw licking and jumping. Both of these are considered to be supraspinally- integrated responses [22]. In order to evaluate the antinociceptive activity of compounds C1 and C2 mediated by opioid receptors, the hot plate test was carried out using the opioid antagonist naloxone hydrochloride. The p.o. doses for compounds C1 and C2 were chosen based on preliminary studies in our laboratory. Pre-treatment with naloxone (1 mg.kg^-1^), did not reverse the antinociceptive effect of both compounds when the animals were treated p.o. with 100 mg.kg^-1 ^doses (p ≤ 0.001), especially at 60 minutes after challenge, suggesting that these compounds do not possess similar action to morphine or derivatives when evaluated in this assay (Table 1).

**Table 1:** Time elapsed (sec.) for nociceptive response in Hot Plate Test in mice related to the time of experiment.

Treatment	Time elapsed after treatment (means ± SD)/ Increase in response time compared to control (%)									
	
	N	0 min	30 min		60 min		90 min		120 min	
*Control*	7	3.4 ± 1.2	3.4 ± 1.5	NS	5.2 ± 0.92	NS	7.04 ± 0.75	NS	6.0 ± 0.95	NS

*Morphine 20 mg.kg*^-1^	6	2.07 ± 0.65	6.9 ± 0.76	203%**	8.15 ± 1.52	163.5%*	13.35 ± 4.05	189.6%**	6.8 ± 2.11	NS

*Naloxone 1 mg.kg*^1^/*control*	6	3.0 ± 0.39	4.72 ± 0.77	NS	5.3 ± 0.7	NS	6.25 ± 1.23	NS	6.65 ± 0.9	NS

*Naloxone 1 mg.kg*^1^/*Morphine20 mg.kg*^1^	8	3.6 ± 1.12	4.81 ± 1.42	NS	7.07 ± 2.23	NS	5.2 ± 1.53	NS	6.0 ± 1.27	NS

*C2 100 mg.kg*^-1^	8	3.5 ± 1.0	4.88 ± 2.21	NS	12.35 ± 1.24	237.5%**	11.5 ± 2.77	163.4%**	7.38 ± 0.76	NS

*Naloxone1 mg.kg*^1^/*C2 100 mg.kg*^1^	7	3.4 ± 1.2	4.21 ± 1.8	NS	11.5 ± 2.11	221.2%**	9.41 ± 2.69	NS	6.65 ± 2.8	NS

*C1 100 mg.kg*^-1^	8	3.1 ± 1.2	6.16 ± 2.0	179%**	10.66 ± 2.13	205%**	8.98 ± 1.48	NS	9.2 ± 2.4	153.3%**

*Naloxone1 mg.kg*^1^/*C1 100 mg.kg*^1^	8	3.5 ± 1.3	5.0 ± 1.68	NS	13.42 ± 2.24	258%**	12.06 ± 2.27	171.3%**	6.7 ± 2.12	NS

Statistical Analysis ANOVA

DUNCAN'S Test: *p < 0.05; **p < 0.01

NS: no significant result

Morphine, Naloxone, C1, C2 and vehicle given orally.

## Conclusion

Both i.p. and p.o. treatment with compounds C1 and C2 reduced response in the writhing, Capsaicin, Glutamate, and hot-plate tests demonstrating their contribution to the antinociceptive activity of crude *Pterodon pubescens *Benth extracts.

Compounds, C1 and C2, demonstrated activity in models demonstrative of vanilloid receptors and glutamate peripheral receptors. In the hot-plate model, the antinociceptive activity was maintained when naloxone hydrochloride, an opioid antagonist, was administered prior to sample dosing suggesting that compounds C1 and C2 do not exert their antinociceptive effects in the hot-plate test via opioid receptors.

The findings presented herein also suggested that crude *Pterodon pubescens *Benth extract probably is a mixture of substances with synergistic interactive effect, since the crude extract (300 mg.kg^-1^) with lower concentrations of compounds C1 (11.5%- 34.6 mg. kg^-1^) and C2 (1.5% - 4.7 mg.kg^-1^) presented statistically analogous effects to those of the pure compounds when tested separately (C1 = C2 = 300 mg.kg^-1^) with p.o. administration in the writhing experimental model.

Further studies will be undertaken to establish the mechanisms of action for compounds C1 and C2. The synergistic interactions will be evaluated employing the Isobole method [23].

## Methods

### 4.1. Plant Material

Seeds were collected in Pedregulho (SP) and São Carlos (SP) cities, in March 2004. Prof. Dr. Jorge Yoshio Tamashiro from IB-UNICAMP (Department of Botany) identified the plant species. A voucher specimen was deposited in the State University of Campinas (UEC) Herbarium, under number 1398.

### 4.2. Compound Isolation

Freeze-dried Seeds (100 g) were ground prior to use in a Stephen mill (model UM 40) and extracted with dichloromethane at room temperature yielding 32% extractable. A portion of the crude dichloromethane extract (32 g) was chromatographed over silica gel (192 g) and eluted sequentially with hexane [F1] (3 × 150 ml); hexane/ethyl acetate 5% [F2] (3 × 150 mL); hexane/ethyl acetate 15% [F3] (3 × 150 mL); ethyl acetate (3 × 150 mL) [F4]; methanol (3 × 150 mL) [F5]. The crude extract fractions were monitored by thin layer chromatography (TLC), visualized with anisaldehyde reagent (50 mL acetic acid, 0.5 mL sulfuric acid and 0.5 mL anisaldehyde) followed by heating at 110°C. Similar fractions were grouped according to their thin layer chromatography profile. Antinociceptive assay indicated that fractions F3 and F4 (10 g) showed activity and they were successively chromatographed by CC on silica-gel (70-230 mesh) (5 × 60 cm) providing 6α, 7β-dihydroxyvouacapan-17β-oate methyl ester and geranylgeraniol with spectral data (FTIR, ^1^H and ^13^C NMR data) in accordance with those reported previously [7].

### 4.3. Chromatographic analysis

The GC/MS analysis was carried out using a HP-6890/5975 system equipped with a J&W Scientific DB-5 fused capillary column (25 m × 0.2 mm × 0.33 m). Temperature program: 60°C (5°C min^-1^) - 300°C (10 min.), injector 250°C, detector 300°C. Helium was used as carrier gas (0.7 bar, 1 ml min^-1^). The MS were taken at 70 eV. Scanning speed was 0.84 scans s^-1^, from 40 to 550. Sample volume was 1 μl. Split: 1:40.

Quantification of 6α, 7β-dihydroxyvouacapan-17β-oate methyl ester and geranylgeraniol in crude *P. pubescens *extract were obtained by internal standard method [24] using butylphtalate as internal standard with authentic 6α, 7β-dihydroxyvouacapan-17β-oate methyl ester and geranylgeraniol samples. Analysis determined 1.6% of 6α, 7β-dihydroxyvouacapan-17β-oate methyl ester (C2) and 11.6% of geranylgeraniol (C1).

### 4.4. High-resolution eletrospray ionization mass spectroscopy (HRESI-MS)

HRESI-MS was recorded on a Q-Tof Mass Spectrometer (Micromass - U.K.) using direct infusion of a 10 μL.min^-1 ^MeOH + 0,1% formic acid solution and ionization by electrospray in the positive ion mode. Major operation conditions were as follow: capillary voltage of 3.5 kV, source temperature of 100°C, desolvation temperature of 100°C and cone voltage of 35 V.

### 4.5. Animals

Male Swiss mice with 25-35 g body weight were kept at 25 ± 2°C in 12 h light-dark cycles (light phase started at 7:00 am) maintained (10 animals per cage) with water and food ad libitum, at least for 7 days prior to assays. Animals were fasted 12 hours prior to oral administration of compounds, in order to avoid possible pharmacokinetic interactions. Separate groups of mice were used for each analgesic test and route of administration, and animals were used only once in experiments. Studies were carried out in accordance with current guidelines for the veterinary care of laboratory animals [25] and were performed under the consent and surveillance of Unicamp's Institute of Biology Ethics Committee for Animal Research (766-1).

### 4.6. Drugs

All drugs and compounds C1 and C2 were diluted in vehicle made of Tween^80 ^1% (Sigma-Aldrich, U.S.A) in saline solution 0.9% (NaCl diluted in distilled water). Reagents (capsaicin and glutamic acid) diluted in phosphate buffer solution (PBS, pH 6.8). The following drugs and reagents were used: pentobarbital (Cristália- Brazil), acetic acid, capsaicin, glutamic acid, naloxone hydrochloride (Sigma-Aldrich, U.S.A) and morphine hydrochloride (FHC, Brazil).

### 4.7. Evaluation of locomotor activity

The open-field test was used to exclude the possibility that the antinociceptive action of compounds geranylgeraniol (C1) and 6α, 7β-dihydroxyvouacapan-17β-oate methyl ester (C2) could be resultant from non-specific disturbances in the locomotor activity of the animals. The ambulatory behavior was assessed in an open-field test as described previously [17] with few changes. The apparatus consisted of a plastic box measuring 45 × 45 × 20 cm, with the floor divided into 9 equal squares (15 × 15 cm). The number of squares crossed with all paws (crossing) was counted in a 3-min session. Mice were treated intraperitoneally (i.p.) with compounds C1 and C2 (30 mg.kg^-1^, i.p.), pentobarbital (35 mg.kg^-1^) or vehicle 30 min beforehand. Results expressed as mean ± S.E.M. of 4 animals per group.

### 4.8. Writhing test

The writhing test was carried out as described by Koster et al [26] with few changes. Groups of mice (*n *= 8) were treated orally (p.o.) or i.p with vehicle (10 mL.kg^-1^) or compounds geranylgeraniol (C1) and 6α, 7β-dihydroxyvouacapan-17β-oate methyl ester (C2) using four doses 10, 30, 100, and 300 mg.kg^-1 ^to determine ED_50_. Dose-related results do not need positive control. Writhing was induced by an i.p. injection of 0.8% acetic acid solution (0.1 mL.10 g^-1^), 30 or 60 min after treatment (i.p. and p.o. respectively). After injection of the acetic acid solution, the numbers of writhings (abdominal constrictions) were cumulatively counted over 15 minutes, for nociception evaluation. Data represent the average of the total writhing observed per dose concentration.

### 4.9. Capsaicin Test

The procedure used was according to Santos and Calixto [27] with few changes. Different groups of animals (n = 8) were treated p.o. with compounds C1 and C2 (300 mg.kg^-1^), morphine hydrochloride (20 mg.kg^-1^), or vehicle, or i.p. with compounds C1 and C2 (10, 30, 100, 300 mg.kg^-1^) or vehicle. No need to use positive control to determine ED_50_. After 60 or 30 minutes (respectively), 50 μL of capsaicin (1.6 μg.paw^-1 ^prepared in PBS) was injected in the ventral surface of the right hind paw. The time that the animals spent licking the injected paw, for the first 5 minutes post capsaicin injection, was recorded with a chronometer and considered as indicative of nociception.

### 4.10. Glutamate test

The glutamate test was carried out as described by Ellson et al [28] with adaptations. Different groups of animals (n = 8) were treated i.p. with compounds C1 and C2 (10, 30, 100 and 300 mg.kg^-1^) (dose- related to determine ED_50_). No need to use positive control to determine ED_50_. Animals received 20 μL of glutamate solution (glutamic acid in PBS) 30 min after compound treatment, injected intraplantar (i.pl.) in the ventral surface of right hind paw (10 μmol.paw^-1^), and were observed individually for 15 min following glutamate injection. The amount of time spent licking the injected paw was chronometered and was considered as indicative of nociception.

### 4.11. Hot Plate Test

The hot plate test was performed according to Woolfe and Mac Donald (1944) [29]. Each animal group (*n *= 16) were treated p.o. with compounds C1 (100 mg.kg^-1^), C2 (100 mg.kg^-1^), morphine hydrochloride (20 mg.kg^-1^), or vehicle, and a half of these groups (*n *= 8) were pre-treated (20 min) p.o. with naloxone hydrochloride (1 mg.kg^-1^) or vehicle (oral administration was used to reduce the stress caused by injection), before thermal algesic stimulation. Mice were placed on the hot plate which was kept at 56°C ± 0.1°C, and the reaction time was noted by observing either the licking of the hind paws, jumping, or the rotation movements at 30, 60, 90, and 120 minutes after administration. For this test, the animals were selected 24 hs before using the same algesic stimulation (cut off 20 sec.).

### 4.12. Statistical Analysis

All results were submitted to one way analysis of variance (ANOVA), considering as critical level p ≤ 0.05 to evaluate significant difference between the control and treated groups, followed by Duncan's Test, using StatSoft^® ^software.

## Authors' contributions

HMS (PhD student) carried out pharmacological studies on antinociceptive experimental models developing new techniques in the laboratory and drafted the manuscript; LS (Master student) carried out Isolation of 6α, 7β-dihydroxyvouacapan-17β-oate methyl ester and geranylgeraniol and gave assistance with pharmacological studies in antinociceptive experimental models; CD (Master Student) carried out initial assays with pharmacological studies with anti-inflammatory experimental models; RAFR carried out the large scale production of authentic samples of 6α, 7β-dihydroxyvouacapan-17β-oate methyl ester and geranylgeraniol; IMOS carried out Analytical support for 6α, 7β-dihydroxyvouacapan-17β-oate methyl ester and geranylgeraniol quantification; JEC as co-supervisor, participated in the design of the study; JYT carried out plant identification; MNE and EC were responsible for HREIMS experiments; MAF Supervisor, conceived design of study, structure elucidation, discussion of pharmacological results and coordination. All authors read and approved the final manuscript.
